# Development and automation of a test of impulse control in zebrafish

**DOI:** 10.3389/fnsys.2013.00065

**Published:** 2013-10-10

**Authors:** Matthew O. Parker, Dennis Ife, Jun Ma, Mahesh Pancholi, Fabrizio Smeraldi, Chris Straw, Caroline H. Brennan

**Affiliations:** ^1^School of Biological and Chemical Sciences, Queen Mary University of LondonLondon, UK; ^2^School of Engineering and Materials Science, Queen Mary University of LondonLondon, UK; ^3^School of Electronic Engineering and Computer Science, Queen Mary University of LondonLondon, UK

**Keywords:** automation, zebrafish, impulse control, 5-choice serial reaction time, screening, mutagenesis

## Abstract

Deficits in impulse control (difficulties in inhibition of a pre-potent response) are fundamental to a number of psychiatric disorders, but the molecular and cellular basis is poorly understood. Zebrafish offer a very useful model for exploring these mechanisms, but there is currently a lack of validated procedures for measuring impulsivity in fish. In mammals, impulsivity can be measured by examining rates of anticipatory responding in the 5-choice serial reaction time task (5-CSRTT), a continuous performance task where the subject is reinforced upon accurate detection of a briefly presented light in one of five distinct spatial locations. This paper describes the development of a fully-integrated automated system for testing impulsivity in adult zebrafish. We outline the development of our image analysis software and its integration with National Instruments drivers and actuators to produce the system. We also describe an initial validation of the system through a one-generation screen of chemically mutagenized zebrafish, where the testing parameters were optimized.

## Introduction

Deficits in impulse control are fundamental symptomatological, and possibly aetiological, factors in a number of neuropsychiatric disorders (APA, 2013). Although the neuropsychological correlates of impulsivity are widely reported (Trifilieff and Martinez, 2013), our understanding of its molecular and cellular basis is relatively limited. Gaining a more advanced understanding of the aetiology and general molecular basis of impulse control would allow the development of individualised treatments and potentially prophylactic interventions for a number of disorders. Although genome-wide association (GWAS) and copy-number variant (CNV) studies are increasing our knowledge of the heritability of impulse control-related disorders (Lesch et al., 2010; Vrieze et al., 2012; Ebejer et al., 2013), ethical constraints and practical difficulties associated with human studies have meant that animal models have gained popularity (Winstanley et al., 2010). With this comes the added benefit of testing specific hypotheses relating to causal mechanisms (Belin et al., 2008; Dalley et al., 2011).

Zebrafish are fast becoming one of the most popular model systems in developmental biology, and their utility as a model in behavioral neuroscience is beginning to be realised (Neuhauss et al., 1999; Blaser and Gerlai, 2006; Levin et al., 2007; Egan et al., 2009; Kyzar et al., 2012; Parker et al., 2013). In order to maximise reliability of data and increase throughput, it is essential that automated testing equipment is developed. This will not only help to ensure good reliability between laboratories and experimenters, but also help to standardise tests and create convergent validity of assays. One of the most exciting prospects for zebrafish research is that this species is amenable to forward genetic screening (Driever et al., 1996; Darland and Dowling, 2001; Ninkovic and Bally-Cuif, 2006). However, endophenotypes that predict psychiatric disorder such as differences in impulse control often are of only very small effect, and as such large numbers of animals are needed in order to identify these in screens. Automation is clearly necessary to facilitate this and exploit the full extent of this species' utility in this regard.

In order to ascertain the suitability for using zebrafish as a model to study the genetic basis of impulse control, robust, repeatable and reliable tasks must be developed so that different laboratories can make contributions to our knowledge. One such task in rodents is the commonly used 5-choice serial reaction time task (5-CSRTT) (Carli et al., 1983; Robbins, 2002; Bari et al., 2008). This is a continuous performance test during which the subject is required to detect the presence of a briefly presented stimulus (usually a light) in one of 5 holes at the rear of the testing chamber. Correct responses (i.e., a nose-poke into the correct hole within a specific time-window) results in food being delivered at the opposite end of the chamber. Errors of omission (e.g., failure to nose-poke within the time window) or errors of commission (responding in the incorrect hole or anticipatory responses) are non-reinforced, or punished (usually 5-s time-out). In the rodent task, this procedure will typically be carried out on a daily basis for many weeks until the animal has reached stable performance. This task has been used to measure aspects of attention and executive performance through manipulation of stimulus exposure length and the addition of distracters. Also this task has proved very useful in testing aspects of impulsivity, operationalised in terms specifically of premature or anticipatory responding (i.e., responding prior to the stimulus onset). Rates of anticipatory responses can be reduced by catecholaminergic transporter blockers such as methylphenidate and atomoxetine, drugs which in turn ameliorate impulsivity in human patients with attention-deficit hyperactivity disorder (ADHD) (Dalley et al., 2011).

We have previously reported the development of a manual version of this task for adult zebrafish (Parker et al., 2012). In addition, there is evidence that larval zebrafish with a transient knock-down in the latrophilin gene (lphn3.1; associated with attention-deficit hyperactivity disorder [ADHD] in humans) may show a type of motor impulsivity that can be reduced by drugs of known efficacy in humans with ADHD (Lange et al., 2012). In this paper, we outline the technical development and automation of the 5-CSRTT as a measure of impulsivity for adult zebrafish, and describe data from a first generation (F_1) screen of N-ethyl-N-nitrosourea- (ENU) mutagenized fish as a validation of the equipment. The reason for including mutagenized fish in this study is that this represents a population that would be expected to show very wide variability in their response patterns as they have a number of functional mutations. In addition, one of the main reasons for using zebrafish for this kind of work is their genetic tractability and the facility to use them for forward genetic phenotype screening. As such, it is important to explore whether sufficient F_1 mutagenized fish are able to perform the task.

## Materials and methods

### Animals

The fish used for the validation of the equipment were 90 4-month-old first-generation ENU mutagenized (Mullins et al., 1994) Tübingen long-fin (kindly donated by Dr F. van Eeden, University of Sheffield, UK), reared in our aquarium facility according to published protocols (Westerfield, 2000). The fish arrived at our facility as embryos (~48 hpf). Prior to testing, the fish were kept in groups of ~10 on a 14:10 light:dark schedule. The temperature of the aquarium was maintained at 26–28°C. Once 4 months old, the fish were moved to our behavioral testing room, and housed in pairs for the duration of the validation experiment. We carried out all experimental work under local ethical guidelines and under the Animals (Scientific Procedures) Act, 1986.

### Design of behavioral testing environment

**Figure 1:**
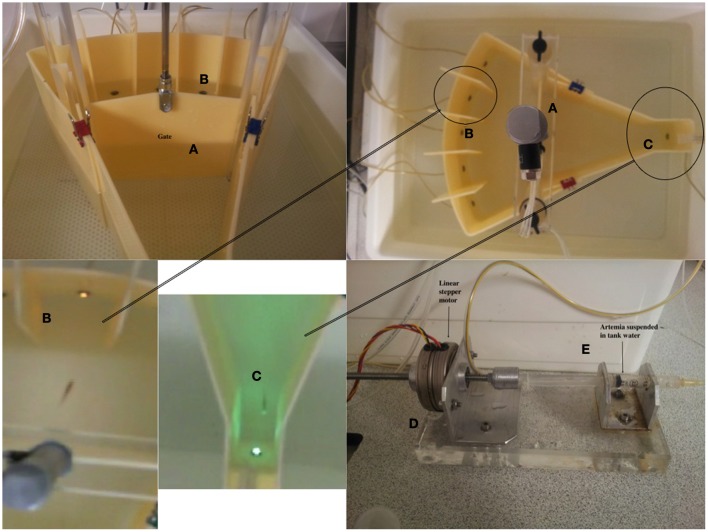
**Figure displays the testing unit and the constituent parts. (A)** The pneumatic gate mechanism. **(B)** The stimulus light area. The stimuli were 5 white LEDs. **(C)** The food delivery area and magazine. This comprised a green LED to act as a stimulus to signal food availability. **(D)** Food was delivered via activation of a linear stepper motor driving the plunger of a 1.5 ml plastic syringe, **(E)**. The food (liquidized bloodworm and brine-shrimp) was delivered to the fish through 1 mm latex catheter tubing.

We designed the testing environment from a combination of commercially acquired and custom-built parts. The entire length of the testing unit was 36 cm, split into two halves by the gate (21 cm from food area to gate, 15 cm from gate to stimulus areas. The external tank (W × L × H: 42 × 49 × 15 cm) was purchased commercially (Ikea, UK). The base was constructed from 10 mm clear cast acrylic and drilled to fix two uprights to support the gate mechanism. The base was profiled to replicate a void which had the same profile as the external perimeter of the testing unit (Figure 1). This void would allow animal waste and surplus food substrate to sink to the bottom. Slots were made in the base to allow for water flow.

A white plastic mesh was placed on top of the base with two holes to allow the two acrylic uprights to pass through. This mesh again allowed animal waste and surplus food to pass through and enhanced the color contrast for the camera system. A pair of clear cast acrylic uprights were fixed in place via stainless steel screws through the base of the unit. These uprights were used to support the sliding gate mechanism and to locate the two halves of the tank profile. Three slots in each post were added to allow for this.

The perimeter of the testing unit and the internal gate were constructed of opaque (ivory) acrylic as this minimised external distractions for the fish being tested, but also avoided reflections within the unit. We had observed in previous pilot studies that having a transparent perimeter resulted in the fish 'pecking' at the side of the tank, and this clearly affected performance in the task. The sliding gate (ivory cast acrylic) was placed into two inner slots and an acrylic bar was placed on the top of the uprights, fixed by stainless steel screws. The gate took less than 1-s to raise/lower. This acrylic bar had, in the center, a tapped (threaded) hole which corresponded to a pneumatic cylinder. There was adjustment both on the thread in the acrylic bar and in the connecting link between ram and sliding door. This allowed the cylinder, via a control system, to raise and lower easily.

The stimulus-area of the tank was curved to allow visual detection of all of the stimuli, five white super-bright light emitting diodes (LEDs), from anywhere in the tank. The food delivery area was tapered to form a small square compartment (the food magazine) in which was located a green LED (the food stimulus) and the food delivery tube (1 mm latex catheter tubing). The entire perimeter of the shaped tank was produced from ivory cast acrylic. The profile ends of the testing unit were machined to length with holes to allow for the use of sealed LED light sources. Dividers were required to fit between the LEDs on the curved profile end of the testing unit, and these were made cast acrylic sheet with a slot to slide over the curved wall. Finally, the feeding unit comprised an acrylic machined clip, which slotted onto the wall of the food area, and a delivery unit which was located outside the tank unit on an acrylic base. The two are connected by catheter tubing. In order to deliver food, we used a linear stepper motor and a 2 ml syringe, both held in line by alloy supports (Figure 1). The stepper motor produced a rotary output which advanced the screw thread, thus forcing the plunger into a syringe that administered the food. The food we used for training was a mixture of liquidized bloodworm mixed with brine-shrimp. Finally, no adhesives or sealant were used, and the use of standard tanks meant there was no need to seal any mechanical item.

### Camera-based fish detection

**Figure 2:**
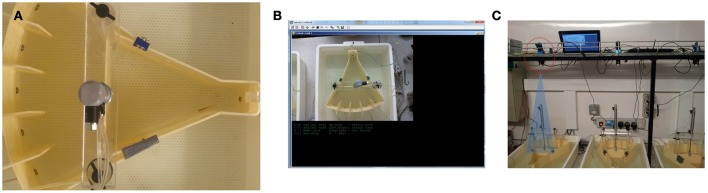
**Camera-based fish detection. (A)** Screen shot of fish in tank. **(B)** User interface of camera-based detection software **(C)** The set up of the testing room and camera position. The camera was located 50 cm above the testing tanks and each was controlled by a separate netbook computer.

Zebrafish were detected using a standard web-cam (Windows LifeCam HD). The camera acquired black and white video at a resolution of 640 × 360 pixels from a distance of ~50 cm, at a frequency of 50 frames per second (fps). Focus was manually set, and the depth of field was sufficient to cover the limited range of water depths (about 10 cm) in the tank. At the available resolution and under ambient illumination, a fish appears as a dark streak of about 30 pixels in length and 5 pixels in width, with practically no detail visible—though a human observer can generally tell the difference between the head and the tail. The latter was often blurred in the images due to motion (See Figure 2).

The apparatus were located in a room with ambient lighting at tank level measured at 160 lx. This meant that during detection, the illumination was not strictly controlled. For example, shadows and reflections were common place, and fish movement and the gate mechanisms in the tank generated ripples in the water. Also, the white LEDs used as stimuli in the tank were sufficiently bright to trigger automatic exposure adjustments in the camera, with consequent changes in the luminance of the image background. Background subtraction was found to be unreliable under these conditions, as was confirmed by using a commercially available tracking system.

We started from the premise that the fish was darker than the tank background, and that detection was only required in relatively small areas of the testing unit in correspondence with the stimuli and the feeding area (Figure 1). Within these areas, all the above illumination changes can be approximated as uniform and would lead to a very low variance of intensity level when the fish is not present (the bottom of the tank being uniformly white). Therefore, we detect the fish using frame-by-frame computation of the variance of the pixel intensity values within the regions of interest, that is then compared against a threshold. The interface displays the variance within each region on a continuously updating graph, which provided a user-friendly method for the operator to set the threshold interactively for each region (the threshold may depend on various factor such as the size of the fish, intensity of illumination, and size of the region). Thresholds were adjusted at the beginning of the experiment and then left unaltered. In order to limit spurious detections, a Schmidtt-trigger mechanism (a circuit that converts an analog input signal to a digital output signal) was employed: a fish is detected in a target region when the variance exceeds the operator-set threshold, and is deemed to have left the area when the variance decreases to below 80% of such threshold. Only then can a new detection be triggered. With such system, sufficient accuracy (Figure 2) can be achieved even on a frame-by-frame basis, without the use of time integration. In order to integrate with the LabView system (see below for details) the fish detection system created temporary files in the LabView working directory, which were serially searched and deleted by LabView as appropriate.

### Hardware development and labview program

Custom made software was written in LabView 2009 (National Instruments, Austin, TX). This software was responsible for delivering food, switching lights on-and-off and moving the gate up and down, and was all controlled via a 95-channel digital USB I/O card (NI-USB-6509, National Instruments, Austin, TX). TTL digital signals were used to control the solenoid valves on the pneumatic gate, and linear actuator for the food delivery through a series of optical couplers (Optocoupler, RS Components, UK). As stated above (see Figure 1), food was delivered through a syringe, the plunger of which was pushed by linear stepper motor (Linear Stepping Actuator 88N, RS Components, UK). The amount of food was determined by the number of pulses sent to the stepper motor drive board (Unipolar Stepper motor drive board, RS Components, UK). The gate system was controlled by a stainless steel piston (see above and Figure 1) driven by pneumatic solenoid valves (RS Components, UK).

**Figure 3:**
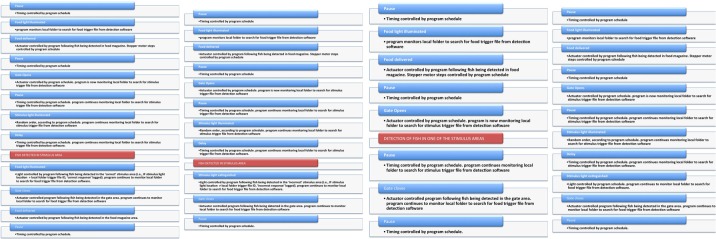
**Simplified workflow for LabView program**. This figure shows the workflow of the LabView program during the course of training on the 5-CSRTT. The panels (**Left** to **Right**) depict the workflow (from the first trial) for trials with correct, incorrect and anticipatory responses, and omissions.

**Figure 4:**
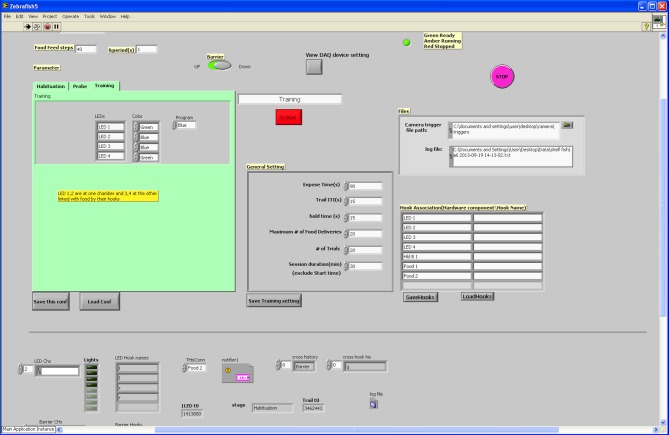
**User interface for LabView program**. The user-interface for the LabView programme was designed to be flexible according to user requirements. We were able to change testing parameters easily prior to each session.

The I-O card was controlled via LabView (National Instruments, UK). In the program, the lighting of the LEDs and the opening of the gate were initially performed according a user-defined session schedule. Subsequent actuator control (lights being extinguished, gate closing, food delivery, etc.) was monitored constantly by the camera-based fish detection program (see above for details). In the LabView program, all events (i.e., the fish entering one of the areas on the testing unit) were monitored constantly as well, and once an event occurred, the detection log file was deleted to allow new event logging. The action performed upon each of the events occurring depended on the session schedule. Actions included LED ignition state change, food delivery (steps on the stepper motor) or gate state change. Figure 3 displays a simplified logical workflow of the LabView program. Figure 4 shows the user interface.

### Validation of system: screen of mutagenized zebrafish

We carried out an initial validation of this system using the first generation offspring of pairings between an ENU-mutagenized male and wild-type (i.e., non-mutagenized) female zebrafish. The fish were grown up in our aquarium facility for 4-months prior to testing. The validation was designed to examine the typical performance parameters of fish in this procedure, as well as examine acquisition and attrition rates. All fish were trained according to the following schedule (for details relating to the equipment, please see Figure 1):

**Week 1**: *Habituation*. Prior to the start of training, the fish are placed in the testing tanks for 30-min undisturbed each day for 1-week. The lights remain on at all times during this time, and food is delivered according to a pre-defined schedule. Here, we delivered food every 30-s following entry to the food magazine area but did not collect any data.

**Week 2**: *Magazine training*. During the second week, the fish were trained to associate the magazine light with food delivery. In this condition, the fish was confined to the food delivery end of the testing environment, with the gate closed. The magazine light was illuminated for up to 10-s, with a 30-s gap between illuminations, during which time the fish could enter the magazine area and receive food. This procedure continued for five 30-min sessions.

**Week 3**: *Stimulus light training*. The fish were initially held in the food delivery area of the tank. The gate was raised after 1-min, revealing the five stimulus areas, all with their lights illuminated continuously. Entry into any of the stimulus apertures resulted in the light being turned off, and the magazine light being illuminated. Re-entry to the food magazine resulted in the gate being lowered, confining the fish to the food delivery area, and food being delivered on entry to the food magazine. After a pause of 20-s, the next trial began. This procedure continued for five 30-min sessions.

**Week 4–8**: *5-CSRTT - 5-s PSI*. Starting in the 4th week, the fish began 5-CSRTT training. Initially, the fish was held in the food delivery area for one minute. The gate was then raised revealing the five stimulus apertures. There was then a delay of 5-s (the pre-stimulus interval; PSI), after which one of the five lights was illuminated for 30-s. Entry to the correct stimulus aperture (i.e., the aperture with the light) during the 30-s stimulus presentation resulted in the magazine light being illuminated. The fish could then swim back to the food delivery area, the gate closed as it passed through, and food was available in the magazine. There was then a delay of 20-s, after which the subsequent trial began as before. Entry to the wrong aperture during the stimulus presentation was called an “incorrect trial,” and resulted in the lights being switched off, and confinement to the food area (with no food) for 20-s. Entry to any of the apertures prior to the light being switched on was called an “anticipatory response,” and again resulted in the lights being switched off, and confinement to the food area (with no food) for 20-s. Finally, if the fish did not respond within the 30-s of the light being illuminated, this again resulted in the lights being switched off, and confinement to the food area (with no food) for 20-s.

**Week 9–11**: *5-CSRTT - 10-s PSI*. During weeks 9–11, the procedure was exactly the same as during weeks 4–8, except that the PSI was increased to 10-s.

Latencies were measured from the start of the stimulus being illuminated. Calculation of general testing parameters were completed in the following way:

$$\begin{array}{l} \;accuracy=\text{correct}/(\text{correct}+\text{incorrect})\\ anticipatory=\text{early}/(\text{correct}+\text{incorrect}+\text{early})\\ \;omissions=\text{omissions}/(\text{correct}+\text{incorrect}+\text{early}+\text{omissions}) \end{array}$$

### Analysis and statistical treatment of data

All data were analysed using the statistical program R (www.r-project.org). Performance data on the 5-CSRTT were fitted to restricted maximum likelihood (REML) general or generalized linear mixed effects models (package lme4 in R; Bates and Maechler, 2009. For magazine training and stimulus light training, the data were fitted to generalized linear mixed models (poisson distribution). For 5-CSRTT training, data were fit to linear mixed effects models. In both cases, the fixed effect was training day (5-levels for magazine and stimulus light training; 20-levels for 5-CSRTT training) with fish ID as the random effect. When we compared the last four sessions of phase 1 and the first four sessions of phase 2, linear mixed effects models were fitted with phase (1 vs. 2) as the fixed effect and ID again as the random effect. Denominator degrees of freedom and subsequent ‘*p*’ values were estimated using the Satterthwaite approximation (Satterthwaite, 1946). Where significant differences were established, *post-hoc* Tukey tests were carried out to further characterise the effects. Data summaries are presented as mean ± sem unless otherwise indicated.

## Results

**Figure 5:**
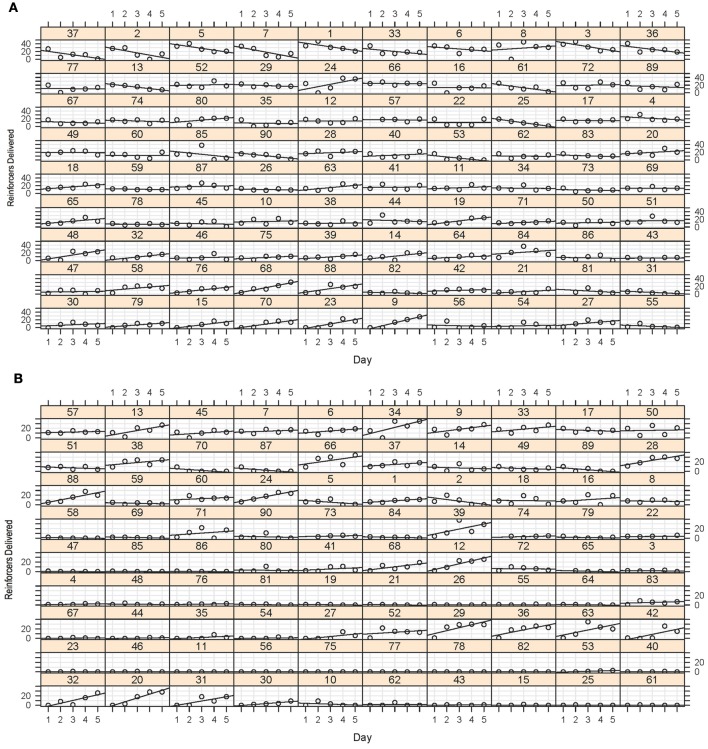
**(A)** Magazine training data. This array displays the number of entries to the food magazine during the magazine light illumination during the five sessions for each of the 90 fish. Although some of the fish showed some evidence of a positive least-squares (LS) regression slope of entries during the sessions, this was certainly not the case for all fish, with many showing a flat or even a negative slope as a function of session. **(B)** Stimulus light training data. Again, although some of the fish showed a very clear increase in number of reinforcers obtained during the sessions, many of the fish did not appear to increase their preference. This did not, however, predict their performance in the later stages of training.

**Figure 6:**
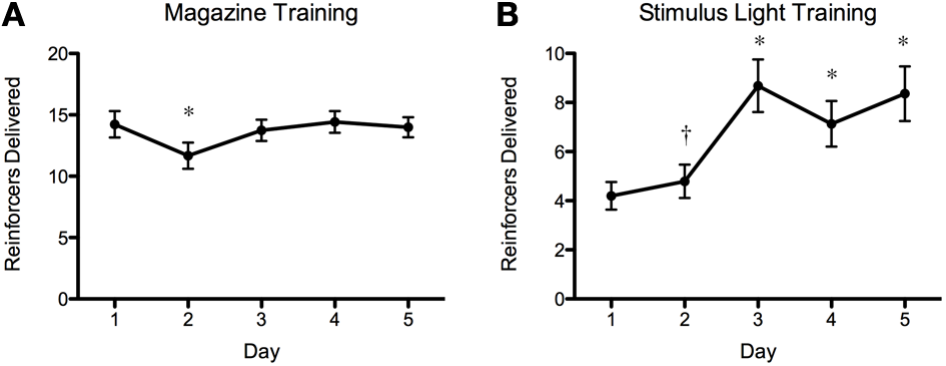
**Magazine training and stimulus light training summary plots**. As above, there was an overall increase in number of reinforcers gained during stimulus light training **(B)**, but this was very variable between individual fish (Figure 5). There was a decrease in the number of reinforcers delivered during magazine training on day 2 **(A)** but no other differences during this phase. ^*^p < 0.01 (*N* = 90; *Post-hoc* Tukey test); † *p* < 0.05 (*N* = 90; *Post-hoc* Tukey test).

The results of the screening experiment showed that although there were clear individual differences in learning there were less consistent patterns of learning for most of the fish in the magazine training (Figure 5A) than in the stimulus light training (Figure 5B) phases of the experiment. During magazine training there was little evidence that the fish increased their entries to the food magazine during the light exposure periods over the course of the five training days. We fitted the data to a generalized linear mixed model (poisson regression) with number of entries to the magazine as the response variable, day as the fixed effect and ID as a random effect, and found that there was a significant reduction in responses on day 2, but no difference on any other days, χ^2^ = 32.23, *p* = 1.717^−06^ (Figure 6A). During stimulus light training there appeared to be a more steady increase in the number of reinforcers received during the course of the five exposure days. We fitted the data to a generalized linear mixed model (poisson regression) with number of food deliveries as the response variable, day as the fixed effect and ID as a random effect, and found a significant increase in food deliveries over the course of the five training days, χ^2^ = 224.42, *p* < 2.2^−16^ (Figure 6B).

During the course of 5-CSRTT training, we had a total attrition rate of 32%. This was due to deaths (*n* = 5) and failing to reach the required level of performance on the 5-CSRTT (a mean of >10 trials per session during training; *n* = 25). Looking at the acquisition curves of the subsequently rejected fish during stimulus light training (excluding those that died during the 5-CSRTT) it was clear that the mean was much lower than those that were kept (rejected = 0.5 vs. retained = 1.33). This initially suggested that there may be some way of detecting poor performing fish based on their slopes during the early (i.e., pre-5CSRTT) training. However, there was no correlation between the individual LS regression slopes during stimulus light training and during 5-CSRTT training [*F*_(1, 59)_ = 0.06, *p* = 0.8, *R*^2^ = 0.001] suggesting significant disconnect between these two training phases.

**Figure 7:**
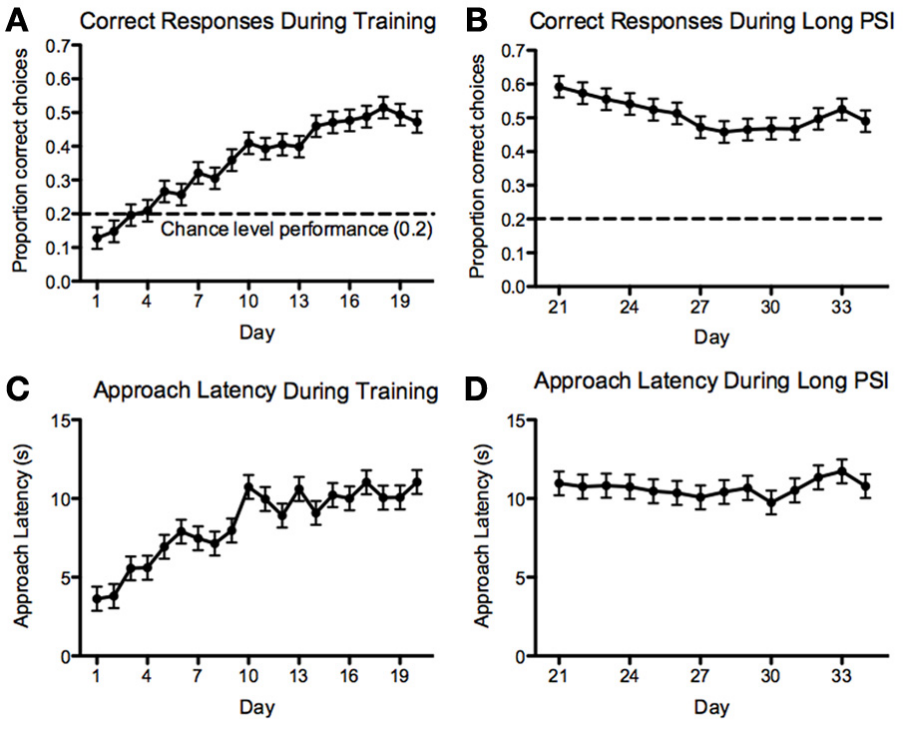
**Correct responses and latency**. Proportion of correct responses **(A,B)** and correct-response approach latencies **(C,D)** during the 5 **(A,C)**- and 10 **(B,D)**-second PSI phases of training on the 5-CSRTT for all fish tested. Note: *N* = 60

**Figure 8:**
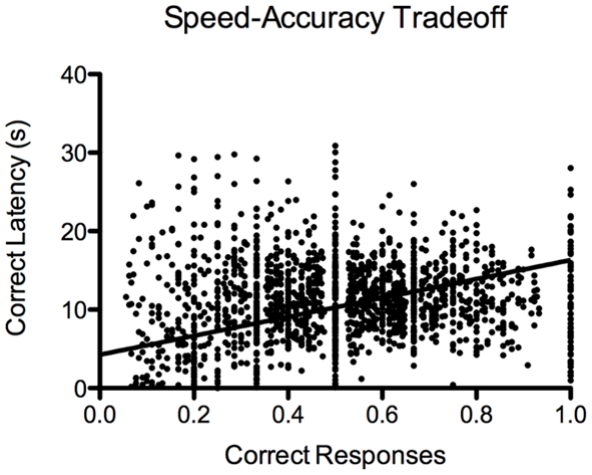
**Speed-accuracy trade-off**. Proportion of correct responses plotted against approach latency (s) during 5-CSRTT training. This provides evidence for a speed-accuracy trade-off during training, where the fish were more accurate when they waited prior to making a response. Note: *N* = 60

For the 5-CSRTT, the data pertain to the remaining 60 fish. We found that the proportion of correct responses increased steadily and significantly prior to introducing the 10-s PSI [*F*_(19, 1136)_ = 19.88, *p* < 2.2^−16^; Figure 7A]. We also found that the correct response latency increased during the course of 5-CSRTT training, again, prior to introducing the 10-s PSI [*F*_(19, 1137)_ = 12.35, *p* < 2.2^−16^; Figure 7C]. We found that the fish showed a significant speed-accuracy trade-off during the course of 5-CSRTT training [*F*_(1, 1176)_ = 490, *p* < 2.2^−16^; Figure 8]. Finally, we tested the difference in correct responses for the final four sessions of the 5-s PSI vs. the first four sessions of the 10-s PSI phase. There was a significant increase in correct responses [*F*_(1, 419)_ = 12.02, *p* = 0.0005818; Figure 7B] but no change in correct response latency [*F*_(1, 419)_ = 0.39, *p* = 0.53; Figure 7D].

**Figure 9:**
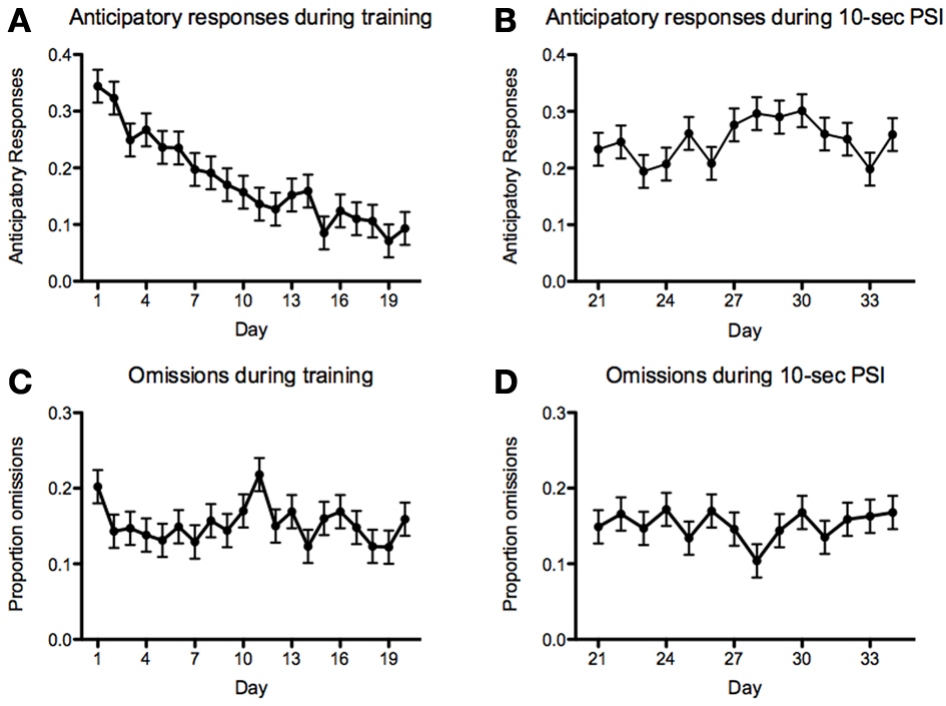
**Anticipatory responses and omissions**. Proportion of anticipatory (i.e., responding prior to the onset of the stimulus) responses **(A,B)** and omissions **(C,D)** during the 5 **(A,C)**- and 10 **(B,D)**-second PSI phases for all fish tested. Note: *N* = 60

With respect to anticipatory responses, we found a steady and significant decrease prior to introducing the 10-s PSI [*F*_(19, 1136)_ = 8.82, *p* < 2.2^−16^; Figure 9A]. We also found that there was a significant increase in anticipatory responses following the introduction of the 10-s PSI (last four sessions of 5-s PSI vs. first four sessions of 10-s PSI: *F*_(1, 418)_ = 88.83, *p* < 2.2^−16^; Figures 9A,B). With respect to omissions, there was a somewhat inconsistent pattern across the course of the 5-CSRTT training during the 5-s PSI phase, with no significant differences between sessions [*F*_(19, 1136)_ = 1.59, *p* = 0.05078; Figure 9C]. We also found no significant difference in omissions following the introduction of the 10-s PSI phase [*F*_(1, 419)_ = 3.07, *p* = 0.08027; Figures 9C,D].

**Figure 10:**
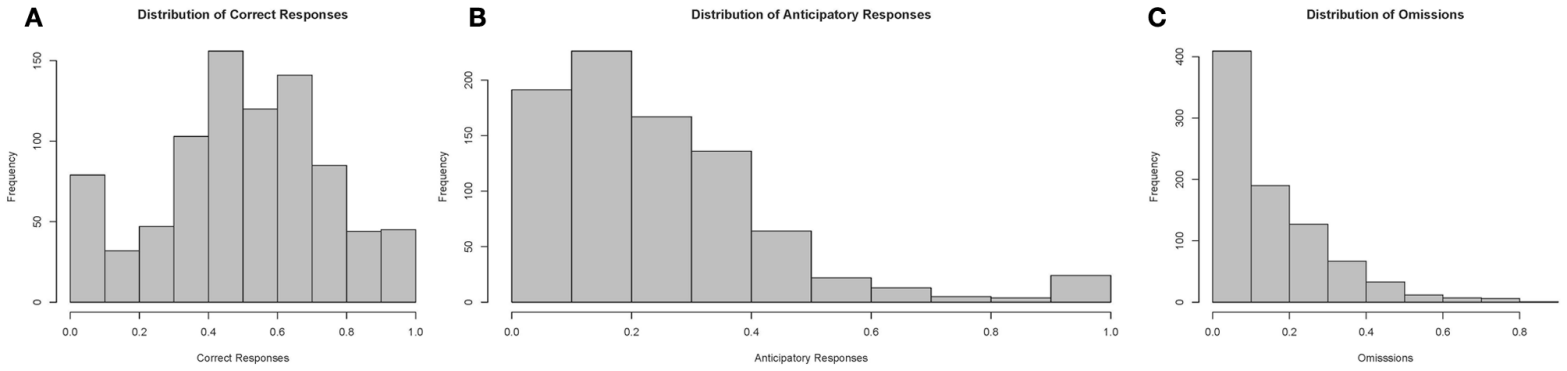
**Histograms of performance parameters**. Correct responses appears to follow an approximately normal distribution, whereas premature responses and omissions were distributed with heavy right skewed tails. Note: *N* = 60. **(A)** displays the distribution of correct responses, **(B)** of anticipatory responses and **(C)** of omissions plotted according to proportion of total responses in a session.

Finally, we examined the distribution of test parameters during the 10-s PSI phase, which are displayed in Figure 10. Correct responses followed an approximately gaussian structure, whereas both anticipatory responding and omissions had heavily right skewed tails, suggesting that only a realtively small proportion of the fish showed very high levels of comission and omission errors during the training process.

## Discussion

In this paper, we have described the development of a fully automated testing system for adult zebrafish, designed to test aspects of impulse control. We have previously demonstrated that zebrafish are able to perform well on a three-choice manual version of this task (Parker et al., 2012), but the automation allows for an increase in throughput, and should increase reliability by decreasing human presence.

Zebrafish have come to the fore as a vertebrate model system for identifying genetic modifiers of developmental disease phenotypes. Our abilty to automate a well established measure of impulse control, and the initial data from mutagenized F_1 fish, underline the potential of this model system to identify genetic modifiers of translationally relevant behavior. ENU mutagenesis involves exposure of male founder fish to a chemical mutagen (ENU) inducing many thousands of mutations into the pre-meiotic germ cells. Founder males are then bred with wild-type females to generate offspring heterozygous for many mutations. The F_1 generation can then be screened for dominant mutations, and subsequent generations (F_3) for recessive mutations. Here we assessed the performance of F_1 mutagenized fish. The overall performance of these fish was pleasing for a number of reasons. First, 60 of the original 90 mutagenized fish used in the study completed training. This is extremely encouraging, as it suggests that a high proportion of these fish will be suitable for phenotype screening for apparently complex behavioral traits such as impulsivity despite the high mutation rate.

Our findings for the mutagenized fish in terms of correct response rate were similar to those previously demonstrated in adult fish on a multi-color discrimination procedure (~60%; Mueller and Neuhauss,^2012^. Approach latencies increased as the training continued, rather than decreased, which was surprising. There was, however, evidence for a significant speed-accuracy trade-off, which may go some way to explaining this pattern. The rates of anticipatory responses decreased during the 5-s PSI training sessions, but increased when we introduced the 10-s PSI, suggesting that this will be a useful tool for examining rates of impulse control in this species. Omission rates were low throughout the training, showing that the timings between trials (20-s) and the amount and type of food delivered was appropriate. Finally, the skewed distributions for anticipatory responses and omissions are to be expected, as this suggests that while most fish generally showed low levels of these performance parameters, there were some fish that showed much higher rates. This is pleasing as it suggests that there are some very high responders that would be suitable for screening for impulsivity phenotypes. As this screen was of F_1 mutagenized fish, the highly skewed distribution for anticipatory responding suggests that there may be some fish carrying dominant mutations that relate to impulsivity in the sample tested here.

The assay as described here requires further validation in terms of translational relevance. One useful way to approach this would be to test the performance either of known mutant zebrafish that would be hypothesised to perform differently on this task, or of zebrafish following pharmacological manipulaiton. For example, the catecholaminergic transporter blockers methylphenidate and atomoxetine are known to reduce impulsivity in both rats and humans with ADHD (Robbins, 2002; Robinson et al., 2007). In our previous study where we examined the performance of adult zebrafish on a manually controlled, 3-choice version of this task (Parker et al., 2012) we did find some evidence that a low dose of amphetamine (0.025 mg/Kg i.p) reduced anticipatory responding during long PSI sessions relative to long PSI sessions following saline injection. This suggested that similar neurobiological processes may control performance on the task. However, that study needs now to be repeated and extended in this automated version, with more doses and a wider variety of pharmacological manipulations.

There are some limitations with the current system, not least of which is that we are unable at this stage to measure attention. The rodent version of this task measures both sustained attention and impulsivity (Robbins, 2002). Specifically, it is able to measure sustained attention by varying the duration of the stimulus presntation (shorter durations require greater attention). Owing to the time it takes for the fish to swim into the stimulus aperture, we have not been sucessful in training the fish with shorter durations. For example, in a pilot study with the present apparatus, we reduced the stimulus duration to 5-s in pilot studies, but the fish were unable to perform at this level (high omission rates, low correct response rate, data not shown). It is unclear at this stage why this is: in our previous study (Parker et al., 2012) we found that adult zebrafish were able to perform well with a 5-s stimulus duration in a 3-choice version of this test. To move forward with assay design, the present procedure could be refined to allow the testing of aspects of attention in adult zebrafish. This could be approached by systematically manipulating the number and duration of stimuli to increase attentional load.

We encountered some difficulties during development, in particular with the camera system. For future development we are designing a new tracking system using infra-red cameras and LEDs. If other groups are considering implementing this system, we would recommend using infra-red to reduce difficulties with tracking owing to inconsistent ambient lighting. For future development of the system, we will also look more closely at the magazine training sessions, as the data from these were inconsistent. In addition, the overall rate of attrition was high (~30%). It is unclear why this is, as it is apparently not related to initial pre-training performance; there was no correlation between pre-training and 5-CSRTT learning slopes. For future studies, we will examine in more detail the drop-off in performance during 5-CSRTT training to try to reduce attrition rates.

One of the great benefits of using zebrafish in neuroscience is the ability to use larvae for high-throughput screening (e.g., phenotypes) and front-line drug discovery (Brockerhoff et al., 1995; Bang et al., 2002; Pardo-Martin et al., 2010). Although there is some evidence that larval zebrafish may be used for high-throughput screening of phenotypes relating to hyperactivity and motor impulsivity (Lange et al., 2012), it is unlikely that impulsivity, as measured in our assay, can be modeled at such an early developmental stage. However, a useful first step may be to screen larvae for evidence of hyperactivity/impulsivity and test identified lines using our system, as adults. It is also plausible that we may be able to identify phenotypic correlates in larvae that would predict adult behavior.

In summary, we have described the construction and automation of a version of the 5-CSRTT for adult zebrafish. The fish detection software and LabView programme described here, as well as plans to develop the physical system, will be made available to other labs on request. In this paper, we have provided initial data pertaining to performance characteristics on the task by ENU-mutagenized adult zebrafish, which by nature should show wide variability in response patterns. These data should provide a useful starting point for other laboratories interested in aspects of impulse control in terms of offering some anticipated data.

### Conflict of interest statement

The authors declare that the research was conducted in the absence of any commercial or financial relationships that could be construed as a potential conflict of interest.
